# Late-onset megaconial myopathy in mice lacking group I Paks

**DOI:** 10.1186/s13395-019-0191-4

**Published:** 2019-02-21

**Authors:** Giselle A. Joseph, Margaret Hung, Aviva J. Goel, Mingi Hong, Marysia-Kolbe Rieder, Noam D. Beckmann, Madhavika N. Serasinghe, Jerry E. Chipuk, Parvathi M. Devarakonda, David J. Goldhamer, Paulina Aldana-Hernandez, Jonathan Curtis, René L. Jacobs, Robert S. Krauss

**Affiliations:** 1 Department of Cell, Developmental, and Regenerative Biology, Icahn School of Medicine at Mount Sinai, One Gustave L. Levy Place, Box 1020, New York, NY 10029 USA; 2 Graduate School of Biological Sciences, Icahn School of Medicine at Mount Sinai, One Gustave L. Levy Place, Box 1020, New York, NY 10029 USA; 3 Department of Genetics and Genomic Sciences, Icahn School of Medicine at Mount Sinai, One Gustave L. Levy Place, Box 1020, New York, NY 10029 USA; 4 Department of Oncological Sciences, Icahn School of Medicine at Mount Sinai, New York, NY 10029 USA; 5 Department of Molecular & Cell Biology, University of Connecticut, Storrs, CT 06269 USA; 6 Department of Agricultural, Food and Nutritional Science, University of Alberta, Edmonton, Alberta T6G 2E1 Canada; 7 Present address: Novartis Institutes for BioMedical Research, 181 Massachusetts Ave, Cambridge, MA 02139 USA

**Keywords:** Pak kinase, Skeletal muscle, Myopathy, Mitochondria, Choline kinase

## Abstract

**Background:**

Group I Paks are serine/threonine kinases that function as major effectors of the small GTPases Rac1 and Cdc42, and they regulate cytoskeletal dynamics, cell polarity, and transcription. We previously demonstrated that Pak1 and Pak2 function redundantly to promote skeletal myoblast differentiation during postnatal development and regeneration in mice. However, the roles of Pak1 and Pak2 in adult muscle homeostasis are unknown. Choline kinase β (Chk β) is important for adult muscle homeostasis, as autosomal recessive mutations in *CHKβ* are associated with two human muscle diseases, megaconial congenital muscular dystrophy and proximal myopathy with focal depletion of mitochondria.

**Methods:**

We analyzed mice conditionally lacking Pak1 and Pak2 in the skeletal muscle lineage (double knockout (dKO) mice) over 1 year of age. Muscle integrity in dKO mice was assessed with histological stains, immunofluorescence, electron microscopy, and western blotting. Assays for mitochondrial respiratory complex function were performed, as was mass spectrometric quantification of products of choline kinase. Mice and cultured myoblasts deficient for choline kinase β (Chk β) were analyzed for Pak1/2 phosphorylation.

**Results:**

dKO mice developed an age-related myopathy. By 10 months of age, dKO mouse muscles displayed centrally-nucleated myofibers, fibrosis, and signs of degeneration. Disease severity occurred in a rostrocaudal gradient, hindlimbs more strongly affected than forelimbs. A distinctive feature of this myopathy was elongated and branched intermyofibrillar (megaconial) mitochondria, accompanied by focal mitochondrial depletion in the central region of the fiber. dKO muscles showed reduced mitochondrial respiratory complex I and II activity. These phenotypes resemble those of *rmd* mice, which lack *Chkβ* and are a model for human diseases associated with *CHKβ* deficiency. Pak1/2 and Chkβ activities were not interdependent in mouse skeletal muscle, suggesting a more complex relationship in regulation of mitochondria and muscle homeostasis.

**Conclusions:**

Conditional loss of Pak1 and Pak2 in mice resulted in an age-dependent myopathy with similarity to mice and humans with *CHKβ* deficiency. Protein kinases are major regulators of most biological processes but few have been implicated in muscle maintenance or disease. *Pak1/Pak2* dKO mice offer new insights into these processes.

**Electronic supplementary material:**

The online version of this article (10.1186/s13395-019-0191-4) contains supplementary material, which is available to authorized users.

## Background

Skeletal muscle health is an important determinant of quality of life. In addition to its pivotal roles in movement, breathing, and temperature regulation, muscle is a highly metabolic tissue [1]. Therefore, proper skeletal muscle homeostasis is not only necessary to preserve optimal muscle mass and strength for movement, but also to prevent adverse systemic effects [2, 3]. Homeostatic maintenance of individual myofibers and muscle tissue is a complex process that requires coordination of many intracellular signaling pathways and structural elements. Defects in such processes result in a variety of skeletal muscle diseases, including muscular dystrophies and congenital myopathies.

Muscular dystrophies are progressive muscle wasting diseases generally characterized by centrally nucleated myofibers, endomysial fibrosis, and necrosis [4]. The most common dystrophies are caused by mutations in components of the dystrophin-associated glycoprotein complex, which links extracellular, laminin-rich matrices to the actin cytoskeleton [5, 6]. Consequently, the structural integrity of myofiber plasma membranes is compromised, increasing susceptibility to contraction-induced injury [7]. Many congenital myopathies result from genetic defects in the contractile and structural proteins of muscle and are defined by distinctive ultrastructural changes or by defects in muscle metabolism [8].

Two muscle diseases, megaconial congenital muscular dystrophy (MDCMC; OMIM #602541) and proximal myopathy with focal depletion of mitochondria (PMFDM; OMIM #600706), were recently shown to be allelic conditions caused by homozygous or compound heterozygous mutations in *CHKB* (encoding choline kinase β) [9]. Patients with these diseases display an unusual and distinctive phenotype: highly enlarged, interfibrillar “megaconial” mitochondria prevalent in the periphery of myofibers, with depletion of mitochondrial activity in central regions [10]. Individuals diagnosed with MDCMC had early-onset muscle wasting and mental retardation, whereas those with PMFDM had later-onset, non-progressive muscle weakness and were cognitively normal [11, 12]. The phenotype of mice lacking Chkβ is consistent with these findings. A spontaneous recessive mutation in mice, *rmd*, resulted from an intragenic deletion in *Chkb* [13]. *rmd* mice have an early-onset muscular dystrophy with a rostrocaudal gradient of severity (i.e., the dystrophic phenotype of hindlimb muscles is worse than forelimb muscles). Similar to patients with *CHKB* mutations, *rmd* mice also display megaconial mitochondria in the myofiber periphery with mitochondrial depletion centrally [10]. CHKβ catalyzes the first step in the synthesis of phosphatidylcholine (PC). *rmd* mice have reduced levels of phosphocholine (pCholine; the direct product of Chk) and PC in their hindlimbs, but how these metabolic defects result in megaconial mitochondria is unclear.

Group I p21-activated kinases (Pak1–3) are versatile signaling proteins activated as effectors of the small GTPases, Rac1 and Cdc42, and which phosphorylate a multitude of substrates [14–16]. This positions them as pivotal regulators of many cellular processes, including cell proliferation, migration, and polarity. These processes are mediated by Pak-dependent regulation of cytoskeletal architecture and gene expression. Group I Paks play important roles in skeletal muscle development. In *Drosophila*, embryonic myoblasts require dPak3 (ortholog of mammalian Pak2) to facilitate the cytoskeletal rearrangements necessary for fusion, with a secondary dependence on dPak1 (ortholog of mammalian Pak1) [17]. We have recently shown that, in mice, Pak1 and Pak2 function redundantly to promote myogenesis [18]. Mice lacking Pak1 and Pak2 conditionally in the skeletal muscle lineage were viable but displayed delayed myoblast differentiation, resulting in reduced muscle mass. During differentiation, Pak1/2 are activated as components of a signaling pathway initiated by N-cadherin-mediated cell adhesion and involving the cadherin coreceptor Cdon with its downstream effector, Cdc42 [18–20]. Pak1/2, in turn, appear to promote differentiation by triggering activity of the promyogenic p38α MAP kinase. Expression of Pak1 and Pak2 is relatively high in the skeletal muscle through the first weeks of postnatal life but reduced by 2 months of age. However, their expression was transiently induced following acute muscle injury and they were redundantly required for efficient myoblast differentiation during regeneration [18]. Pak1 was also induced by denervation, playing a role in coupling neuronal activity to muscle gene expression [21].

Many factors that are involved in developmental myogenesis do not play major roles in the maintenance of mature skeletal muscle, and their expression is accordingly downregulated in adult muscle. Expression of Pak1 and Pak2 fits this pattern; however, Pak1 and Pak2 are detectable in adult muscle, albeit at low levels, suggesting they may play a role in muscle homeostasis. Here we report that mice lacking Pak1 and Pak2 conditionally in the skeletal muscle lineage develop a late-onset myopathy, characterized by the presence of megaconial mitochondria. Although later in onset and less severe than the pathology seen in *rmd* mice, the disease phenotype of Pak1/2 mutant mice similarly occurs in a rostrocaudal gradient. These findings reveal an unexpected role for group I Paks in muscle and mitochondrial homeostasis.

## Methods

### Mice

*Pak1*^*−/−*^, *Pak2*^*f/f*^, and *MyoD*^*iCre*^ mice were as described [22–24]. *Pak2*^*f/f*^;*MyoD*^*iCre/+*^ conditional knockout (cKO) mice were crossed to *Pak1*^*−/−*^ animals to generate the *Pak1*^*−/−*^;*Pak2*^*cKO*^ double knockout (dKO) animals as described previously [18]. *Pak1*^*+/−*^;*Pak2*^*f/f*^ animals were used as the control genotype. All mice were maintained on a mixed C57BL/6/FVB background. *Dmd*^*mdx-4Cv*^*(Mdx)* mice were from the Jackson Laboratory (Bar Harbor, ME). All animal procedures were conducted in accordance with institutional guidelines for the care and use of laboratory animals as approved by the Institutional Animal Care and Use Committees (IACUC) of the Icahn School of Medicine at Mount Sinai and the University of Connecticut.

### Evans blue dye assay

Evans blue (Sigma) was diluted to 1% in PBS and sterile filtered before use. Animals were treated by intraperitoneal (IP) injection of diluted Evans blue dye (EBD) at 1% volume relative to body mass. Two-month *mdx* mice were also treated as a positive control for the assay. All mice were sacrificed at 24 h post-injection, and TA and quadriceps were harvested for cryosectioning and imaging as described below.

### Cell culture

C2C12 cells were cultured as previously described [25, 26]. Briefly, cells were maintained as sub-confluent, proliferating cultures in growth medium (GM; DMEM + 15% fetal bovine serum) and passaged as needed using 0.05% Trypsin-EDTA (Gibco). To evaluate differentiation, cells were plated 24 h prior to being transferred to differentiation medium (DM; DMEM + 2% horse serum) at approximately 80% confluence. Cells were assessed over a 3-day differentiation time course.

### Preparation of protein extracts for western blotting

Protein extracts were prepared from quadriceps and brain tissue as detailed elsewhere [18]. Briefly, snap-frozen tissues were homogenized on ice, in RIPA lysis buffer using a loose pestle. Protein lysate was then collected via microcentrifugation. For protein extracts from cells, cold Tris lysis buffer (50 mM Tris-HCl pH 7.5, 100 mM NaCl, 1% Triton-X 100, Protease and Phosphatase inhibitor cocktail) was added directly to the culture dish, and cells were collected by scraping the dish. Cells in lysis buffer were transferred to an Eppendorf tube and incubated for 30 min on ice with periodic agitation. Samples were then microcentrifuged for 30 min at 4 °C, after which the supernatant was transferred to a fresh tube. All extracts were stored at − 70 °C until analysis. Protein concentration was quantified using the Bradford assay kit (Bio-Rad), prior to western blot analysis.

### Western blot analysis

Samples were diluted to desired concentrations with lysis buffer and loading dye and denatured in boiling water for 20 min. The diluted proteins were separated by sodium dodecyl sulfate polyacrylamide gel electrophoresis (SDS-PAGE) on a 10% acrylamide gel (Protogel, National Diagnostics), and subsequently transferred onto an activated PVDF membrane (Millipore). The membrane was then blocked in 5% milk in TBST (1 M Tris-HCl pH 7.5, NaCl, KCl, 0.1% Tween 20 in deionized water) for 1 h at room temperature and incubated with primary antibody overnight at 4 °C. After three washes in TBST, membranes were incubated in the appropriate anti-rabbit or anti-mouse secondary antibodies for 1 h at room temperature.

### Antibodies

Primary antibodies used for western blot were anti-choline kinase α, anti-Mfn2 (Abcam); anti-Drp1, anti-Opa1 (BD Biosciences); anti-Pak1, anti-Pak2, anti-phospho-Pak1(S144)/Pak2(S141), anti-pDrp1(S637), anti-Tom20 (Cell Signaling Technology); anti-choline kinase β, anti-Vdac1, anti-Mfn2 (Santa Cruz); and anti-Gapdh (Ambion). Secondary antibodies used were anti-mouse HRP IgG and anti-rabbit HRP (Cell Signaling Technology). For immunofluorescence, primary antibodies used were dystrophin and Ki67 (Abcam), Pax7 (DSHB), and laminin (Sigma). Alexafluor-488-conjugated wheat germ agglutinin and secondary antibodies (anti-mouse IgG1 Alexafluor-647 and anti-rabbit Alexafluor-488) were from Invitrogen. For references on use of selected antibodies, see [18] (Pak1, Pak2, pPak1/2, GAPDH); [27] (DRP1, pDRP1(S637), Opa1, Mfn1, Mfn2,); [28] (laminin); [29] (Ki67); and [30] (choline kinase α).

### Preparation of frozen tissue sections

TA or quadriceps muscles were isolated and immediately mounted in 10% Tragacanth (Sigma-Aldrich) and flash frozen in chilled 2-methylbutane (Fisher Scientific). Ten-micrometer-thick tissue sections were obtained with a Leica CM3050 S.

### Hematoxylin and eosin stain

Tissue sections were stained as described previously [18]. Tissue was fixed in 4% paraformaldehyde on ice for 10 min and rinsed briefly with water five times. Sections were submerged in Harris modified hematoxylin (Fisher) for 1 min, rinsed five times in water, and then flushed with water for 5 min. Following this, they were quickly rinsed in acid wash solution (70% ethanol + 1% hydrochloric acid in water) three times and again rinsed five times in water. Eosin Y stain (Ricca) was applied for 1 min, and sections were rinsed in water five times. Tissue dehydration was carried out in increasing concentrations of ethanol: 50%, 70%, and 95% for 1 min each; 100% for 3 min; 100% for 5 min; and clearing in xylene (Fisher Scientific) for 3 min. Sections were mounted in Permount (Fisher Scientific).

### Masson’s trichrome stain

Tissue sections were stained using the Masson’s trichrome stain kit (Polysciences, Inc.), following the manufacturer’s protocol with some modifications. Briefly, muscle sections were fixed in Bouin’s solution for 1 h at 60 °C and then rinsed in running tap water to remove the yellow color. Slides were incubated in freshly made Weigert’s iron hematoxylin working solution (equal parts stock solution A and B) for 10 min at room temperature, followed by a rinse in warm running tap water for 10 min, and a quick rinse in distilled water. Biebrich scarlet-acid fuchsin solution was used to stain sections for 10 min, with a quick rinse in distilled water followed by a 15-min incubation in phosphomolybdic-phosphotungstic acid to differentiate. Sections were transferred directly to aniline blue solution for 10 min, rinsed quickly in distilled water and then differentiated in 1% acetic acid solution for 3 min. After a short wash in distilled water, sections were dehydrated by very rapid passes in 95%, then 100% ethanol, and cleared in xylene. Slides were mounted using Permount (Fisher Scientific).

### Modified Gomori trichrome stain

Cryosectioned tissues were brought to room temperature and immersed in acidified Harris modified hematoxylin (Fisher Scientific) for 5 min. Sections were then washed with running tap water until the water ran clear. Slides were incubated in Gomori trichrome stain (12.8 mM Chromotrope 2R (Sigma), 3.7 mM Fast green FCF (Sigma), 2.1 mM phosphotungstic acid (Sigma), 0.2 M acetic acid (Fisher), deionized water, pH 3.4) for 20 min at room temperature. Excess stain was removed with two quick rinses in freshly made 0.2% acetic acid, and slides immediately placed in 95% ethanol. Tissues were then dehydrated in ascending concentrations of ethanol, cleared and mounted as described above.

### Cytochrome oxidase stain

Muscle sections were stained in incubating solution (0.2 M sucrose (Sigma); 1.4 mM 3, 3′ diaminobenzidine tetrahydrochloride (DAB; sigma); 1.4 mM cytochrome C (Sigma); 8 mM catalase (Sigma); dI water; 50 mM phosphate buffer, pH 7.6) for 1 h at room temperature. Slides were then washed with three exchanges of tap water. Tissue dehydration, clearing, and mounting were performed as outlined above.

### NADH-tetrazolium reductase stain

Freshly cryosectioned tissues were used for analysis. Sections were incubated in a 1:1 dilution of β-nicotinamide adenine dinucleotide, reduced disodium salt hydrate solution (NADH; 2.3 mM NADH (Sigma) and 0.05 M Tris buffer, pH 7.6) and freshly made nitrotetrazolium blue chloride solution (NBT; 2.45 mM NBT and 0.05 M Tris buffer, pH 7.6) for 30 min at 37 °C, and then quickly rinsed with three exchanges of water. Unbound NBT was removed with 2-min passes in increasing/decreasing/increasing concentrations of acetone solution: 30%, 60%, and 90%. Tissues were then rinsed in several exchanges of water to remove acetone and mounted in Aquamount (Lerner Laboratories).

### Sirius Red stain

Cryosectioned tissues were fixed in Bouin’s solution at 56 °C in a humidity chamber for 15 min, washed twice in distilled water, and incubated in 0.1% Sirius Red in saturated picric acid (Electron Microscopy Sciences, 26, 357–02) for 1 h at room temperature. Slides were washed twice in 0.5% acetic acid, dehydrated in ethanol, and equilibrated in xylenes for 10 min. Slides were mounted with Permount (Fisher Scientific, SP15–100) and left to dry in the hood overnight. Sirius Red-positive areas were quantified with Image J.

### Imaging

Images of all histological stains were obtained using a Nikon 26 Eclipse TS100 Microscope, coupled with a ProgRes CF cool camera (Jenoptik, Germany) and ProgRes Mac CapturePro software (Jenoptik, Germany).

### Immunofluorescence microscopy and electron microscopy

Pax7 and laminin co-staining on muscle sections was performed as described by Goel et al. [28]. For all other stains, sections were fixed in 4% PFA for 10 min on ice, followed by a gentle rinse with PBS. Permeabilization of tissue was achieved by a 10-min incubation in 0.3% Triton-X 100 in PBS at room temperature. Sections were rinsed in multiple rounds of 0.1% Triton-X 100 in PBS (PBST) and then blocked in 30% goat serum in PBST for 1 h at room temperature. Dystrophin antibody was diluted in blocking buffer, and slides were stained overnight at 4 °C. Following primary antibody labeling, the tissue was carefully washed multiple times with PBST and then incubated with the appropriate Alexafluor secondary antibody diluted in blocking buffer for 1 h in the dark at room temperature. After several washes in PBST, sections were counterstained with DAPI and finally mounted with Fluoromount (SouthernBiotech). Fluorescent images, including EBD, were captured on the Axioimager Z2 microscope (Zeiss) with the Axiocam 503 mono (Zeiss) and Plan-Neofluar 10x/0.3 and Plan-Achropomat 20x/0.8 lenses. The software used was Zen2 Blue edition version 6.1 (Zeiss).

For electron microscopy, animals were perfused intercardially with a prewash of 1% paraformaldehyde (PFA)/PBS at 8 ml/min until cleared and tissue was fixed with 2% PFA plus 2% glutaraldehyde/PBS at the same flow for 10 min. The skin was removed and the animals were immersion fixed in the same solution at 4 °C for 24 h. Electron microscopy was performed by the Microscopy CoRE at the Icahn School of Medicine at Mount Sinai. Morphometric analysis of mitochondria was analyzed with Image J software.

### Mitochondrial complex activity assays

Complex I, II, and IV activities were measured in muscle tissue protein lysates using the Complex Enzyme Activity Microplate Assay Kit (Abcam) specific to each complex. Protein was isolated from the quadriceps of 1-year old animals. Briefly, minced tissue was incubated in 0.05% Trypsin-EDTA (Gibco) for 30 min on ice and agitated every few minutes. The tissue was then collected by centrifugation for 10 min at 4 °C, the supernatant discarded, and the muscle washed in cold mitochondrial isolation buffer (MIB; 200 mM mannitol, 68 mM sucrose, 10 mM HEPES pH 7.4, 1 mM EDTA, 1 mM EGTA, 0.1% BSA) and centrifuged again. The muscle was resuspended in fresh MIB and then transferred to a glass dounce tube on ice. MIB was used to make up the remaining volume, and the tissue was homogenized with approximately 40 passes of the dounce pestle. Tissue homogenate was then transferred to a fresh Eppendorf tube, and detergent supplied with the Abcam kit was added. Samples were incubated for 30 min on ice, and lysate was collected by centrifugation at the appropriate speed and time for the specified assay kit. Protein concentration was determined by BCA assay (Pierce). Once lysate was obtained, the manufacturer’s protocol was followed to determine complex activity. Both MIB and the appropriate complex incubation buffer (Abcam kit) were used as individual negative controls for the assay, whereas bovine heart mitochondria isolate was used as a positive control.

### Lipid analysis

Lipid content (mg/100 g tissue) was assessed in the hindlimb (quadriceps) and forelimb muscle of 1-year-old mice. Choline-related compounds including free choline, phosphocholine (pCholine), and phosphatidylcholine (PC) in samples were determined using the hydrophilic interaction liquid chromatography-tandem mass spectrometry (HILIC LC-MS/MS) using conditions and scan modes as previously described, with minor modifications [31, 32]. The analysis was performed using an Agilent 1200 LC system equipped with an Ascentis Express, 2.7-μm particle size, 150 mm × 2.1 mm HILIC column (Sigma) coupled to a 3200 QTRAP mass spectrometer (AB SCIEX) under turbospray positive mode. The mobile phase A was acetonitrile and B was 10-mM ammonium formate in water at pH 3.0, adjusted using formic acid. The gradient was as follows: 0–0.1 min, 8% B; 0.1–10 min, from 8 to 30% B; 10.1 min, jumped to 95% B; 10.1–22 min, 95% B and then back to 8% B at 22.1 min for column re-equilibrium prior to the next injection, giving a cycle time of 35 min/injection. The flow rate of mobile phase was 400 μl/min for the period from 28 to 34 min and 200 μl/min for all other periods; before 2.5 min and after 22 min, the LC effluent was diverted to waste.

### Statistical analysis

The unpaired Student’s *t* test was used to determine statistical significance and was conducted using GraphPad Prism 7.04 software, except for Fig. 6d, which used a one-way ANOVA followed by Dunnett’s multiple comparison tests (see Figure 6d Legend).

### Analysis of the Genotype-Tissue Expression portal

The Genotype-Tissue Expression (GTEx) project (https://www.gtexportal.org/home/) collects and analyzes human tissues from densely genotyped donors for global RNA expression within individual tissues, allowing treatment of gene expression levels as quantitative traits. Therefore, variations in gene expression that are highly correlated with genetic variation can be identified as expression quantitative trait loci or eQTLs. GTEx portal summary files were downloaded for multi-tissue eQTLs of PAK1 and PAK2. Figures were generated using R version 3.4.4 [33] and the R packages ggplot2 [34]. Data are expressed as normalized effect size (NES). NES is computed as the effect of the alternative (i.e., eQTL) allele relative to the reference allele in the human genome reference GRCh37/hg19.

## Results

### Mice lacking group I Paks do not recover muscle mass with age

**Fig. 1:**
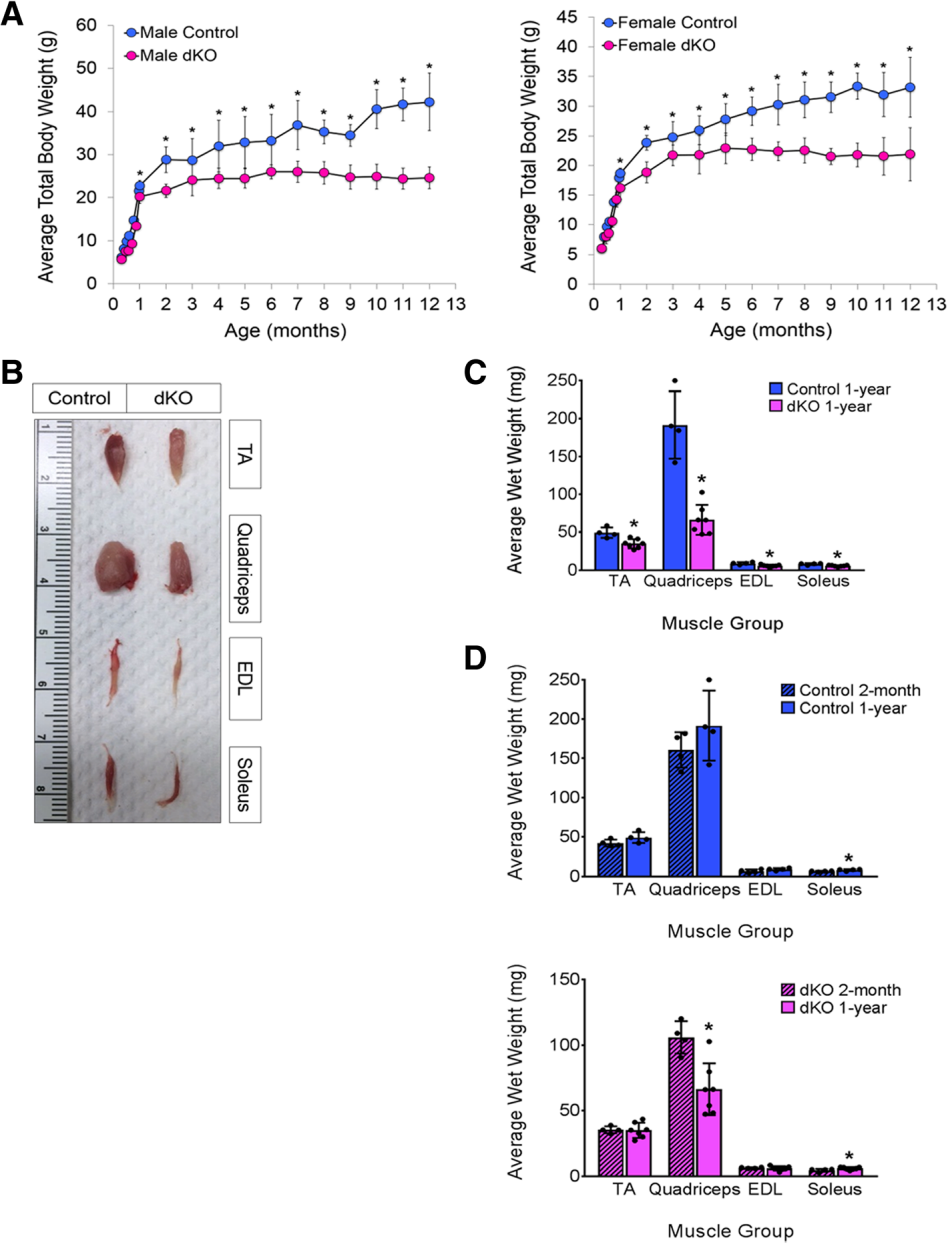
Body and muscle weights are reduced in mice lacking group I Paks. **a** Total body weight of male and female, control and dKO mice, measured at monthly intervals. *n* ≥ 6 mice per sex and genotype. **b** Representative images of selected muscle groups from 1-year-old animals. **c** Wet weights of individual muscle groups in **b**. Control mice *n* = 4, dKO mice *n* = 7. **d** Comparison of wet weights of individual muscles from 2-month- and 1-year-old male control and dKO animals. Control mice, 2-month *n* = 4, 1-year *n* = 4; dKO mice, 2-month *n* = 4, 1-year *n* = 7. Values are means ± SD, *p < 0.05

We previously reported on mice lacking Pak1 and Pak2 conditionally in the skeletal muscle lineage (designated dKO mice) [18]. dKO mice carry a germline mutation of *Pak1* and a conditional mutation of a floxed *Pak2* allele with the *MyoD*^*iCre*^ driver, which is active beginning at the developmental myoblast stage [22–24]**.** The *MyoD*^*iCre*^ driver is very efficient and Pak3 is not expressed in the skeletal muscle of control or dKO mice, so dKO mice essentially lack all group I Pak activity [18]. *Pak1*^*+/−*^;*Pak2*^*f/f*^ animals were used as the control genotype. Two-month-old dKO mice had significantly reduced body weight and muscle mass compared to control animals [18]. Because this phenotype resulted from delayed muscle differentiation, we investigated whether dKO mice normalized their muscle mass given time. The total body weights of male and female animals were measured from birth, at monthly intervals over a 1-year time course. Both male and female early postnatal dKO animals were comparable in weight to controls; however, a significant divergence in weight began at 1 month of age (Fig. 1a). This is in keeping with our prior observations of reduced body mass of 2-month-old dKO mice [18]. Control mice continued to increase in weight with age over the 12-month period. In contrast, dKO animals showed only a slight increase in mass from 2 to 3 months (Fig. 1a). The weight of dKO mice plateaued at 3 months of age, remaining similar until the final measurement at 12 months (Fig. 1a).

At the end of the time course, the wet weights of the tibialis anterior (TA), quadriceps, extensor digitorum longus (EDL), and soleus from control and dKO mice were measured. The mass of all muscle groups from the dKO mice was appreciably lower than their control counterparts, with the quadriceps showing the most obvious difference (Fig. 1b, c, Additional file 1: Figure S1). In fact, although the wet weights of the TA, EDL, and soleus were incrementally higher in 1-year- vs. 1-month-old mice of either genotype, the 1-year-old dKO quadriceps was 30% smaller than that of 2-month-old dKO animals (Fig. 1d). Together, these results indicate that the effect of combined Pak1 and Pak2 loss in muscle continues throughout life, as dKO mice did not recover body or muscle mass with age. Loss of muscle mass, specifically in the quadriceps of older dKO mice, suggested a progressively worsening phenotype. This phenotype was not observed in *Pak1*^*−/−*^ or *Pak2*^*f/f*^*;MyoD*^*iCre*^ mice, nor were any of the myopathic phenotypes described below (data not shown). Therefore, either Pak1 or Pak2 are sufficient for adult muscle maintenance.

### Mice lacking Pak1 and Pak2 develop a late-onset muscle disease

**Fig. 2:**
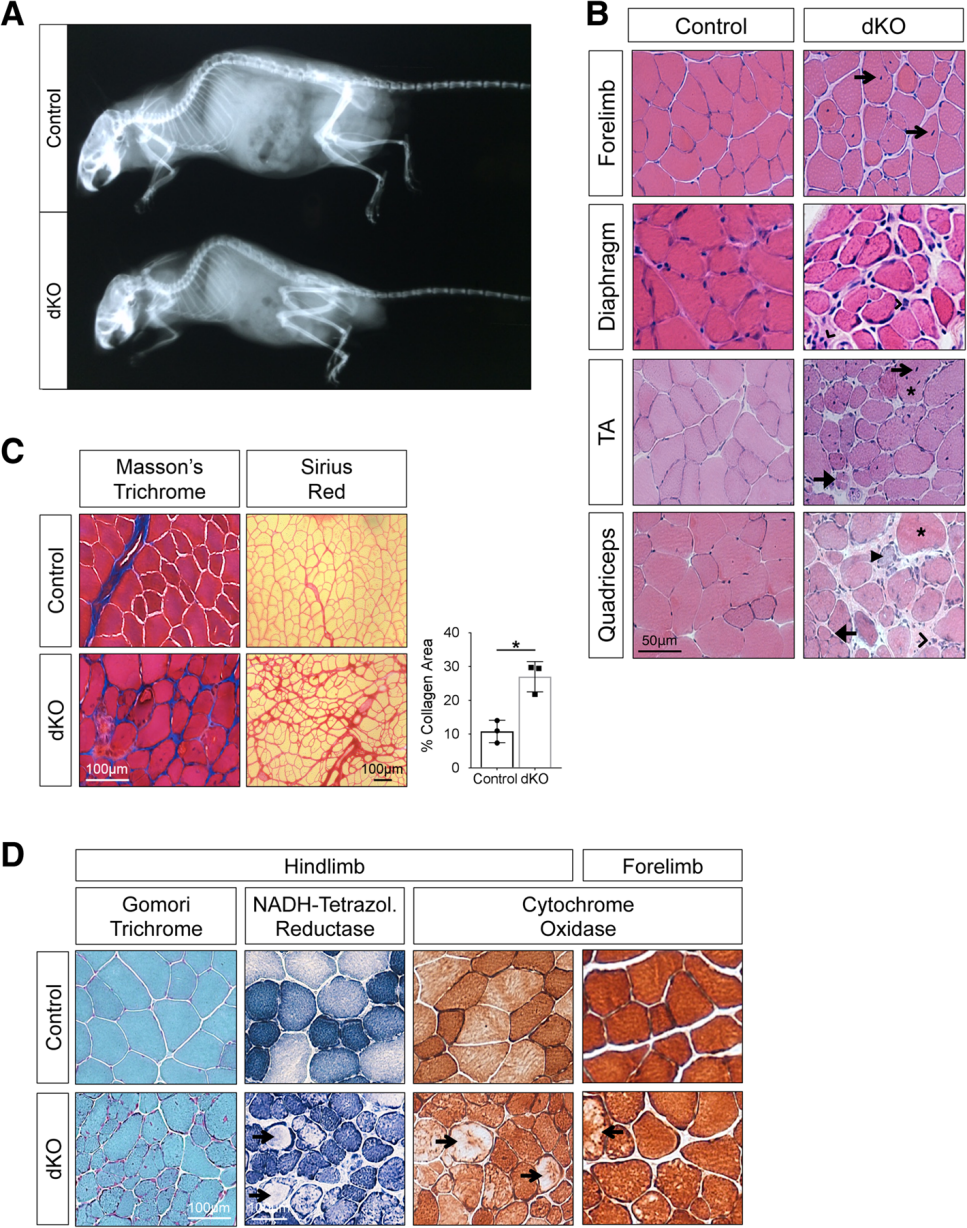
Histopathology of age-related myopathy in dKO mice. **a** Representative X-ray analysis of 1-year control and dKO mice. Spines of dKO mice show abnormal rounding (kyphosis). **b** H&E-stained sections of representative muscle groups from control and dKO animals. Muscles from dKO animals display indicators of disease: central nuclei (open arrow), inflammation (open arrowhead), necrosis (closed arrowhead), hypertrophy (asterisk), and atrophy (closed arrow). **c** Detection of endomysial fibrosis in control and dKO quadriceps muscles by Masson’s trichrome stain (blue) and Sirius Red stain (red) as indicated. The histogram quantifies the Sirius Red-positive area from control and dKO sections. *n* = 3 mice per genotype, with each point representing the average Sirius Red-positive area of five randomly selected sections per mouse. Values are means ± SD, *p < 0.05. **d** Stains for mitochondrial histology as indicated. Gomori trichrome stain for mitochondria (purple) and nuclei (black). Qualitative analysis of complex I enzymatic activity and fiber type distribution by NADH-tetrazolium reductase stain; type I fibers (dark blue) and type II fibers (light blue). Cytochrome oxidase stain for complex IV activity and fiber type distribution; type I fibers (dark brown) and type II fibers (light brown). Arrows indicate focal depletion of mitochondria in dKO myofibers

dKO mice progressively developed a hunched posture, which became prominent at the latter end of the time course. This phenotype was reminiscent of kyphosis, an exaggerated rounding of the spine attributed to weakening of paraspinal and respiratory muscles [35]. X-ray analysis confirmed abnormal spinal curvature in 1-year-old dKO animals compared to age-matched controls (Fig. 2a). All dKO mice presented with kyphosis by 1 year of age. To determine whether the kyphosis was due to pathological changes in the muscle, we performed H&E staining of the forelimb, diaphragm, TA, and quadriceps muscles from 1-year-old control and dKO animals. All muscles from control animals showed normal morphology (Fig. 2b). However, the majority of fibers in the TA and quadriceps of dKO mice had central nuclei (Fig. 2b). Additionally, histological analysis revealed tissue necrosis, as well as a mix of hypertrophic and atrophic fibers. There also appeared to be significant splitting of hypertrophic fibers, which likely contributed to the presence of small, atrophic-appearing fibers (Fig. 2b). These features were most pronounced in the quadriceps. Central nuclei were also detected in myofibers from the forelimb and diaphragm of dKO mice, with myofiber organization more disrupted in the diaphragm than forelimb muscle (Fig. 2b). However, overall, these tissues were not as markedly affected as the TA and quadriceps, suggestive of a phenotypic gradient, with increasing severity from the forelimb to hindlimb. The apparent progressive nature of the muscle disease in dKO mice prompted us to determine the time of onset of the phenotype. As previously described, fibers from 2-month-old dKO mice generally have normal morphology [18]. However, a closer analysis of muscles in these animals revealed that approximately 64% (*n* = 14) had small, infrequent areas that displayed similar features to those seen in older dKO mice (Additional file 2: Figure S2). Therefore, the penetrance and severity of the phenotype increased with age, indicating that the disease was progressive.

To further characterize the pathological phenotype in the dKO muscle, histological analyses were performed on hindlimb tissue sections. Masson’s trichrome and Sirius Red staining revealed significantly enhanced collagen deposition within the endomysium of dKO muscle, a strong indication of fibrosis (Fig. 2c). Modified Gomori trichrome stain is used to diagnose traditional mitochondrial myopathies [36]. Here, dKO fibers displayed punctate, cytoplasmic staining not seen in control muscle (Fig. 2d). Nonetheless, we did not detect the classic “ragged red fibers” that are characteristic of mitochondrial myopathies. Qualitative assays for mitochondrial function with the NADH-tetrahydrozolium reductase (NADH-TR) and cytochrome oxidase (COX) stains reveal two types of information: fiber type distribution and mitochondrial function. The oxidative type I fibers stain comparatively darker than type II fibers in both these assays and usually occur in a mosaic pattern in healthy tissue, as was seen in the controls (Fig. 2d). We observed a loss of distinct fiber type patterning in dKO muscle (Fig. 2d). Moreover, the central areas of many dKO fibers were depleted of staining in both assays (Fig. 2d). Forelimb muscle from dKO mice displayed comparatively fewer myofibers with focal depletion of the COX stain, in keeping with the milder phenotype than the hindlimb dKO muscle (Fig. 2d). Together, these findings indicate that dKO mice develop age-related myopathy with complete penetrance, thus identifying a role for group I Paks in muscle homeostasis. Furthermore, loss of Pak1/2 differentially affects muscle groups, with a rostrocaudal gradient of severity.

### Muscles from dKO mice are not overtly dystrophic

**Fig. 3:**
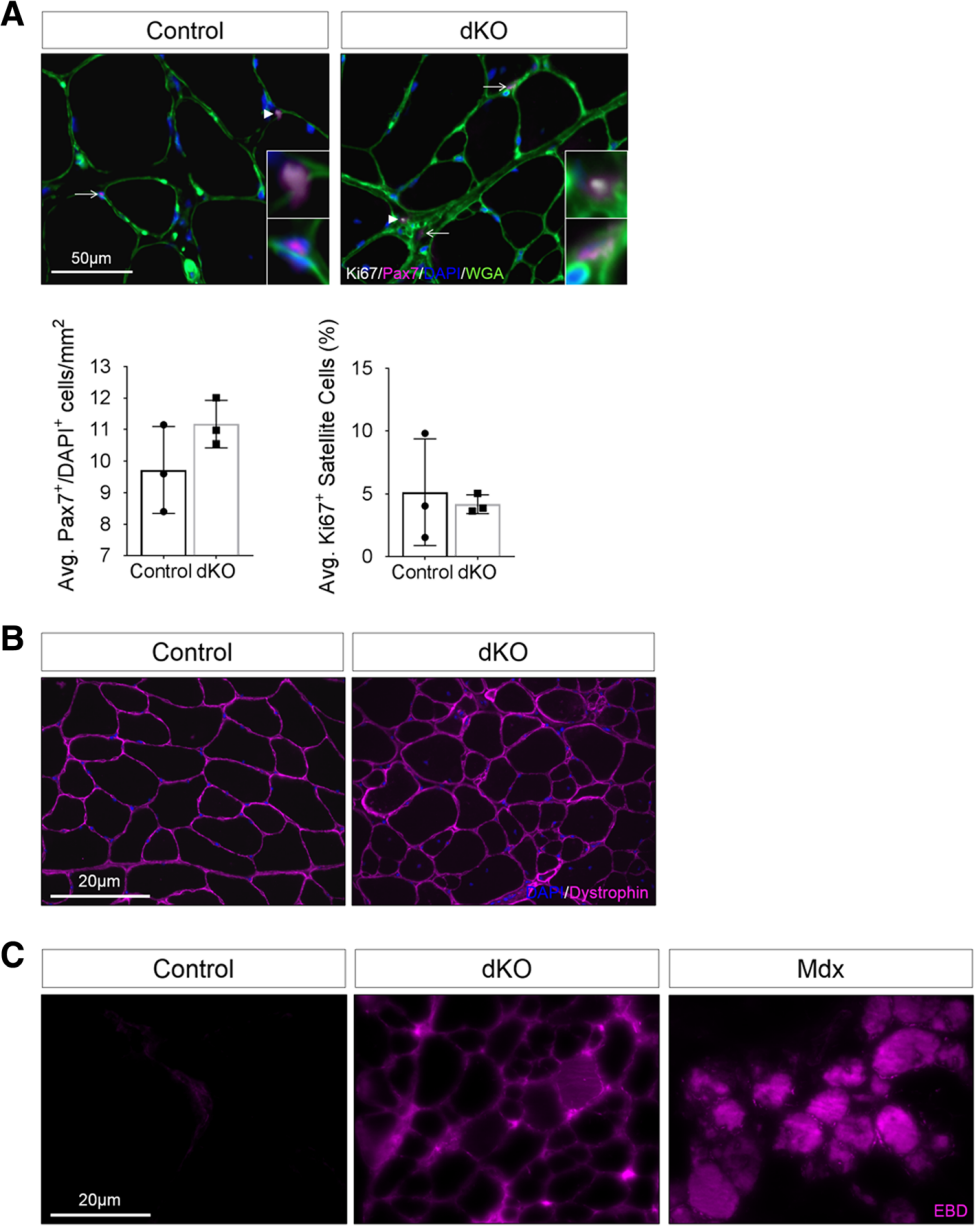
Myopathic dKO muscles are not obviously dystrophic. **a** Analysis of Pax7 and Ki67 expression by satellite cells in TA muscles of 1-year-old control and dKO mice. Pax7^+^ cells are indicated by arrows; Pax7^+^/Ki67^+^ cells are indicated by arrowheads. Individual myofibers are revealed by labeling with wheat germ agglutinin (WGA; green), and nuclei are identified by DAPI (blue). The top panels of the insets are the cells identified by arrowheads, and the bottom panels of the insets are the cells identified by arrows. The histograms quantify the average number of Pax7^+^/DAPI^+^ cells per mm^2^ (left) and the average number of Pax7^+^ cells that are also Ki67^+^ (right). *n* = 3 mice per genotype, with each point representing the average value from 20 randomly selected sections per mouse. Values are means ± SD, *p < 0.05. **b** TA muscles of 1-year-old control and dKO mice immunostained for dystrophin (magenta) and DAPI (blue). **c** Comparative evaluation of myofiber membrane permeability by Evans blue dye (EBD; magenta) uptake in 1-year-old animals. The experiments in **b** and **c** were repeated three times, with similar results

Central nuclei in the myofibers of dKO mice may arise through cycles of degeneration and satellite cell-mediated regeneration, as seen in muscular dystrophies. For example, *mdx* mice, which model Duchene muscular dystrophy, have centrally-nucleated fibers and elevated numbers of satellite cells [37]. Additionally, prolonged regeneration may eventually lead to a reduction in satellite cell number over time. Quantitative IF analyses of Pax7 expression showed a comparable number of satellite cells in 1-year-old control and dKO muscles (Fig. 3a). Additionally, dKO satellite cells remained in their niche, under the basal lamina (Fig. 3a). A similar low percentage (< 5%) of control and dKO Pax7^+^ cells were also positive for the cell proliferation and satellite cell activation marker Ki67 (Fig. 3a). These results argue that, unlike in *mdx* mice [38], satellite cells in dKO mice are not in a myopathy-induced state of activation.

Another hallmark of some dystrophies is the absence or mislocalization of dystrophin from the fiber membrane. Immunostaining for the dystrophin protein in TA muscle sections from 1-year-old dKO and control mice revealed no overt differences in its levels or localization (Fig. 3b). Moreover, assessment of myofiber membrane integrity by the Evans blue dye (EBD) uptake assay demonstrated very infrequent uptake of EBD by dKO fibers, with the majority of the dye signal being trapped between the fibers, indicative of collagen-mediated fibrosis [39] (Fig. 3c). Similar results were obtained with the quadriceps muscle. Despite the poor quality of the dKO muscles, the results indicate that their myofiber membranes were not overtly compromised.

### dKO muscles have megaconial mitochondria

**Fig. 4:**
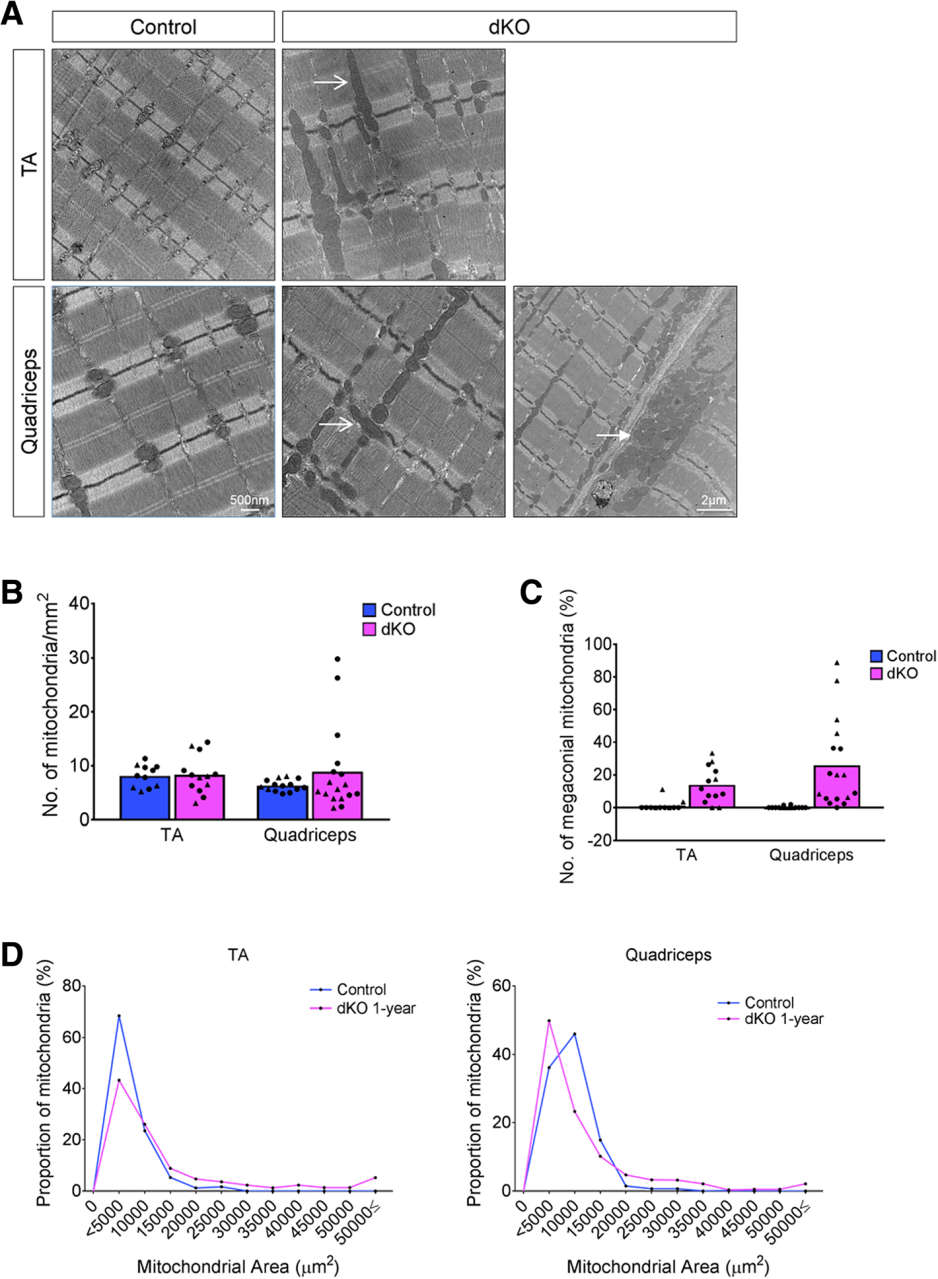
Identification of megaconial mitochondria in dKO muscle. **a** Electron micrographs of longitudinal sections of a 12-month control and dKO TA and quadriceps. Megaconial mitochondria (open arrow) and subsarcolemmal accumulation of mitochondria (closed arrow) are indicated in dKO mice. 500-nm scale bar pertains to all micrographs except the lower right panel, where the indicated bar represents 2 μm. Quantitative analysis of the number of mitochondria per unit area (**b**), the percentage of megaconial mitochondria (**c**), and the proportion of mitochondria of the indicated size (**d**), all as assessed by analyses of electron micrographs. For each genotype, two individual animals are depicted by a black circle or triangle, each point representing the count from randomly selected, independent sections of different myofibers

Because the TA and quadriceps were the most severely affected muscles in dKO mice, we conducted a more refined evaluation of them to gain further insight into their myopathy. Ultrastructural analysis of longitudinal muscle sections by electron microscopy revealed no major differences in the sarcomeres of control and dKO mice, although some areas of dKO muscle showed misalignment of the sarcomeres relative to each other (Fig. 4a). Normally, mitochondria are located within the I-band of the sarcomere (Fig. 4a). Strikingly, dKO muscle presented with grossly elongated (megaconial) mitochondria, in many instances spanning the entire length of the associated sarcomere (Fig. 4a). Furthermore, we observed misoriented mitochondria, elongated 90° from the normal orientation, and occasional subsarcolemmal accumulation of mitochondria in dKO fibers. The average number of mitochondria in either muscle group was similar between genotypes (Fig. 4b). However, dKO muscle had a higher percentage of enlarged, megaconial mitochondria, which were very rare in control muscles (Fig. 4c, d).

**Fig. 5:**
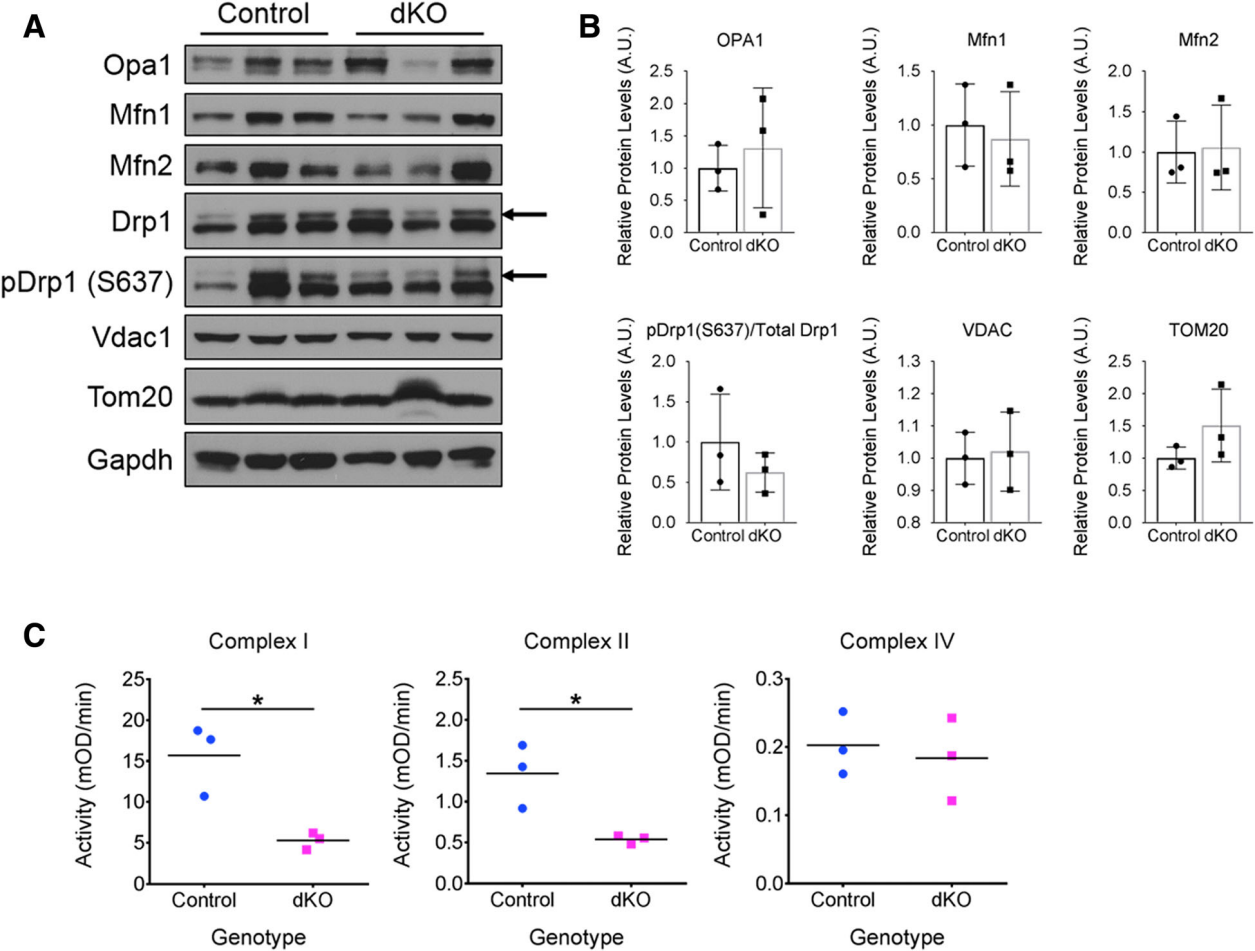
Analysis of mitochondrial protein expression and respiratory complex function. **a** Western blot analysis of mitochondrial proteins in quadriceps muscle extracts from 1-year-old control and dKO mice. Gapdh was used as a loading control. Three mice of each genotype are shown. The arrows indicate Drp and pDrp bands; the bands below are non-specific. **b** Densitometric quantification of the results in **a**; Gapdh was used for normalization. **c** Quantitative analysis of mitochondrial respiratory chain complexes I, II, and IV activity of the hindlimb muscle. *n* = 3 mice per genotype, *p < 0.05

Mitochondrial size is often reflective of the metabolic state of the cell and closely regulated by a balance between fusion and fission of existing mitochondria. Mitofusins 1 and 2 and Opa1 control fusion in the outer and inner mitochondrial membranes, respectively, while Drp1 (and its phosphorylated derivative, pDrp1) controls mitochondrial fission [40–43]. Western blot analysis showed no change in the levels of these regulatory proteins in dKO muscles, despite the profound changes in mitochondrial size (Fig. 5a, b). Additionally, the levels of two outer mitochondrial membrane proteins, Vdac1 and Tom20, were also similar between control and dKO muscles (Fig. 5a, b), suggesting that the total mitochondrial area is not substantially different between control and dKO muscles. A similar observation has been made in *rmd* mice [44], which also display megaconial mitochondria.

We next examined mitochondrial electron transport chain function. Quantitative analysis of the enzymatic activity of complexes I and II of the respiratory chain revealed that activity for both complexes was reduced by > 60% in dKO muscle compared to control muscle (Fig. 5c). In contrast, complex IV activity was not significantly different between control and mutant animals (Fig. 5c). These findings are consistent with the NADH-TR and COX staining results and are strongly indicative of specific mitochondrial dysfunction in dKO muscle.

### Analyses of potential interactions between Chkβ and group I Pak activities

*rmd* mice have a deletion of *Chkβ* and develop a rostrocaudal muscular dystrophy [10, 13], and their phenotype has similarities to patients with autosomal recessive mutations in *CHKB* [10, 13]. As with dKO mice, the hallmark of the *rmd* phenotype is megaconial mitochondria with impaired function, including frequent focal depletion of NADH-TR and COX staining in myofibers [10]. Additionally, both dKO and *rmd* mice show a gradient of myopathic severity from the forelimb to hindlimb muscle [13]. We hypothesized that a mechanistic connection may exist between Chkβ and Pak1/2 that could account for the similarity of the phenotypes. Perhaps Chkβ activity and its phospholipid products are important for Pak1/2 activation, as specific lipids can modulate Pak1 and Pak2 activity [45, 46]. Alternatively, group I Pak activity may regulate Chkβ activity. Pak1/2 consensus phosphorylation sites are not present in Chkβ, making direct phosphorylation unlikely, but the effects of Pak1/2 could be exerted indirectly through phosphorylation of other factors that regulate Chkβ function. To begin to address these questions, we investigated potential links between Chkβ and Pak1/2 in mice and cultured myoblasts.

**Fig. 6:**
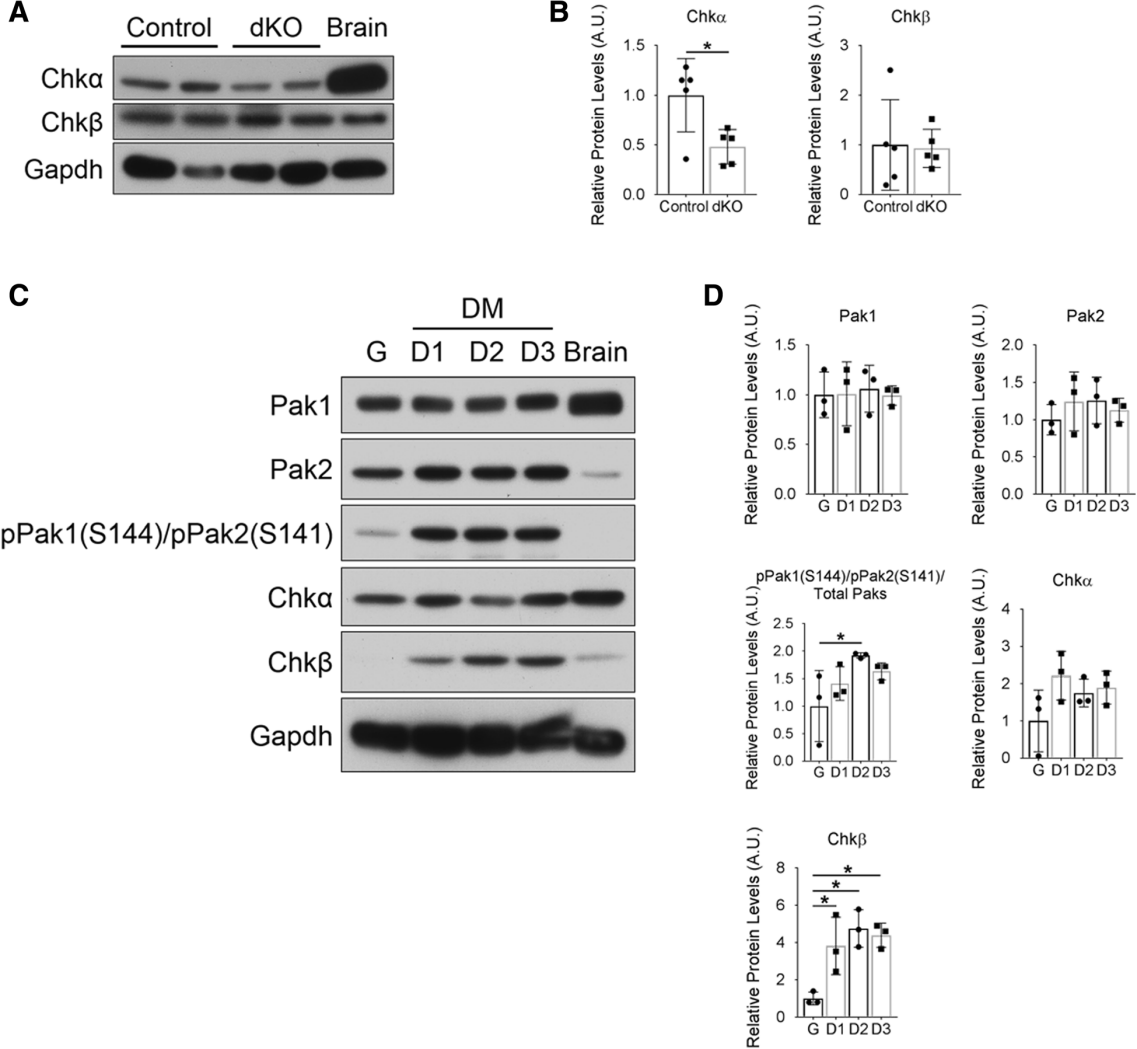
Analysis of choline kinase expression in muscle and C2C12 myoblasts. **a** Western blot analysis of choline kinase (Chk) isoform expression in quadriceps extracts from 1-year-old control and dKO mice. Brain protein lysate used as a positive control for Chk antibodies. Two mice of each genotype are shown. **b** Densitometric quantification of the results in **a** and similar western blots; Gapdh was used for normalization. *n* = 5 mice per genotype, *p < 0.05. **c** Western blot analysis of the indicated proteins in C2C12 cells cultured in growth (G) medium and for the indicated number of days in differentiation (D) medium. **d** Densitometric quantification of the results in **c** and similar western blots; Gapdh was used for normalization. *n* = 3 independent experiments, one-way ANOVA followed by Dunnett’s multiple comparison tests was used for statistical analysis. The means of all groups were compared to growth conditions, *p < 0.05

**Fig. 7:**
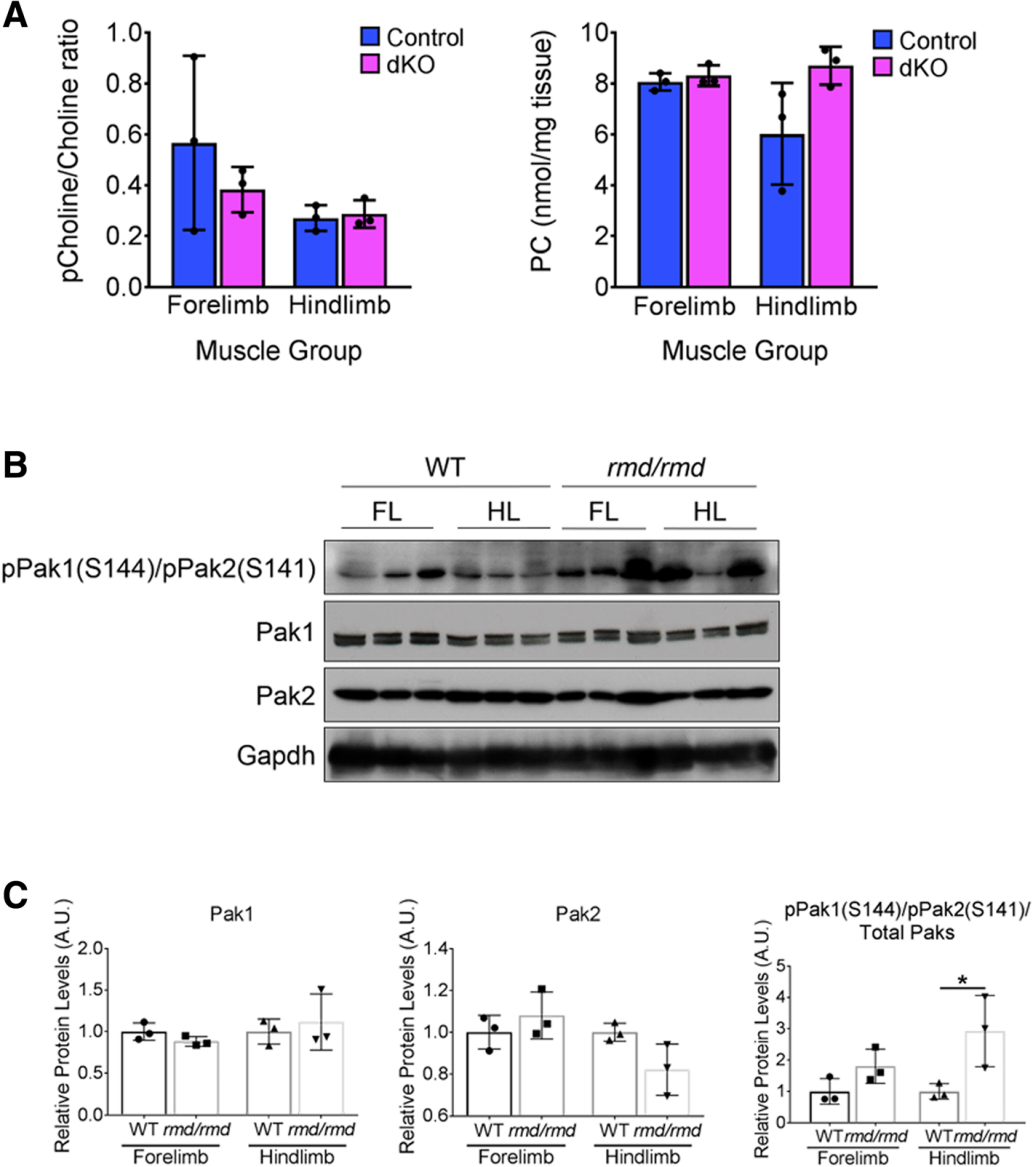
Analysis of choline kinase activity in control and dKO mice and Pak1/2 phosphorylation in control and *rmd* mice. **a** Ratio of phosphocholine (pCholine) to total choline (left graph) and total phosphatidylcholine (PC) levels (right graph) in the forelimb and hindlimb (quadriceps) muscles of three independent 1-year-old control and dKO mice quantified by mass spectrometry. Histograms represent means ± SD. **b** Western blot analysis of activated Pak1 and Pak2 in the forelimb and hindlimb muscle from P11 control and *rmd*/*rmd* mice. For all western blot analyses, 150 μg of protein were loaded per sample. Three mice of each genotype are shown. **c** Densitometric quantification of the results in **b** and similar western blots; Gapdh was used for normalization. *n* = 3, *p < 0.05

Muscle extracts from 1-year-old dKO and control mice were immunoblotted with antibodies for Chkβ and its isoform, Chkα. While Chkβ levels were similar in control and dKO quadriceps muscles, Chkα levels were reduced by ~ 50% in dKO tissue (Fig. 6a, b). Next, we sought to determine Chk expression levels in relation to Pak1 and Pak2 activation during in vitro differentiation of C2C12 myoblasts. Increasing levels of Chkβ correlated with Pak1 and Pak2 phosphorylation in differentiating C2C12 cells, whereas Chkα levels remained unchanged (Fig. 6c, d). To directly assess whether group I Paks are required for Chk activity, we quantified the levels of choline, phosphocholine (pCholine; the direct product of Chk), and PC by mass spectrometry in muscles of control and dKO animals. The ratio of pCholine to Choline and the total PC levels were not significantly different between 1-year-old control and dKO mice in both the forelimb and hindlimb muscles (Fig. 7a). The diminished levels of Chkα seen in dKO muscle (Fig. 6a, b) were therefore insufficient to alter total levels of Chk products. We conclude that group I Pak activity is not a critical regulator of Chk activity.

We next asked whether Chkβ function regulated Pak1 and Pak2 activity. Phosphorylated Pak1 and Pak2 (pPak1/2) are readily detectable in the muscle from postnatal day 11 (P11) control animals [18]. To determine if Pak1/2 activation is affected by genetic loss of *Chkβ*, we assessed pPak1/2 levels in protein extracts from the forelimb and hindlimb muscle of P11 *rmd* mice. Total levels of Pak1 and Pak2 were not different between wild-type and *rmd* mice (Fig. 7b, c). Unexpectedly, hindlimb muscles from *rmd* mice showed a statistically significant increase in pPak1/2 levels (Fig. 7b, c), opposite from our prediction. These data indicate that Chkβ is not required for phosphorylation of Pak1 and Pak2, but its absence may result in feedback mechanisms that elevate signaling through Pak1/2.

## Discussion

We report here that mice lacking Pak1 and Pak2 in the skeletal muscle lineage develop a late-onset myopathy. We previously showed that these animals had a delay in myoblast differentiation resulting in reduced muscle mass; muscles of 2-month-old dKO mice were smaller than controls but had normal morphology [18]. However, by 10 months of age, dKO muscles displayed centrally-nucleated fibers, fibrosis, and signs of degeneration.

Several of the myopathic features of dKO mice bear a strong resemblance to *rmd* mice, which have a mutation in *Chkb* [13]. These include: (1) megaconial mitochondria, (2) central depletion of mitochondrial activity, and (3) a rostrocaudal gradient of disease severity. *rmd* mice are considered dystrophic, but dystrophin was normally localized and only scattered fibers took up EBD [10, 13]. This was also the case for dKO mice, in which dystrophin was appropriately localized and EBD-positive myofibers were rare. There are also clear differences between *rmd* and dKO mice. Onset of muscle disease in *rmd* mice occurs much earlier (weeks vs. months) and is more severe, than in dKO mice. Additionally, functional analyses of mitochondrial energetic function showed that complex I and II, but not complex IV, activities were diminished in dKO muscles, whereas complex I and IV, but not complex II, activities were diminished in *rmd* muscles [44]. We attempted to measure basal and maximal oxygen consumption rates with Seahorse assays on cultured single isolated myofibers from control and dKO mice, but the fibers did not withstand the conditions and underwent lethal contraction (not shown). Such data are not available on fibers from *rmd* mice either. Nevertheless, the most distinctive phenotype of dKO mice is the highly unusual formation of megaconial mitochondria, often with lack of mitochondrial activity centrally, a phenotype shared by choline kinase β deficiency in both humans and mice; we are unaware of other mutations that produce this phenotype in skeletal muscle.

Although megaconial mitochondria are a distinguishing characteristic of both Chkb/CHKB and Pak1/2 deficiency, it is not clear whether they are the cause of the myopathy or a consequence of a different, underlying molecular and cellular defect. Nevertheless, our findings suggest there may be a link between Chkβ and Pak1/2 activities. Consistent with this idea, *Chkb* expression was induced in differentiating cultured myoblasts similar to Pak1/2 activation. However, while pCholine and PC levels were reduced in *rmd* mice and patients with mutations in *CHKB*, they were normal in dKO mice. Additionally, Pak1/2 phosphorylation was not dependent on the presence of Chkβ. In fact, levels of phosphorylated (activated) Pak1/2 were elevated in the hindlimb muscles of *rmd* mice. Together, these results argue against a simple, direct regulatory relationship between Chkβ and Pak1/2 activities.

Although *rmd* mice have lower than normal PC levels in mitochondrial membranes, it is not obvious why this results in megaconial mitochondria. It is possible that Pak1/2 substrates regulate mitochondrial integrity and localization in a manner that converges mechanistically on PC-dependent processes. Mitochondria are specifically localized at I-bands in striated muscle [47]. Transport and localization of mitochondria are regulated by the actin-, microtubule-, and intermediate filament-based cytoskeletons [48]. Regulation of cytoskeletal dynamics is a major function of group I Paks [49, 50]. Pak1/2 may promote mitochondrial integrity and localization through phosphorylation of substrates that regulate one or more cytoskeletal systems important for mitochondrial homeostasis. By this rationale, Chkβ deficiency-induced changes in mitochondrial membrane phospholipid composition could interfere with linkage of mitochondria to cellular cytoskeletal elements. It will be important to identify what are sure to be a multitude of Pak1/2 substrates in skeletal muscle to provide insight into this complex problem.

An alternative possibility is that the megaconial myopathy in dKO mice arises secondarily from defects in a role of group I Paks in insulin-stimulated glucose uptake. Pak1/2 are activated in response to insulin signaling in muscle and are reported to promote insulin-dependent GLUT4 translocation and glucose uptake in this tissue [51–54]. This may occur via phosphorylation of the Arp2/3 complex component p41ARC [55]; indirect regulation of cofilin phosphorylation status by Pak1/2 is also proposed to play a role in this phenomenon [56]. However, Moller et al. have recently reported a systematic analysis of insulin-mediated glucose uptake in the series of Pak mutant mice under study here (including dKO mice) and concluded that group I Paks are largely dispensable for regulation of glucose uptake in muscle [57]. Therefore, defects in glucose clearance and metabolism seem less likely to underlie the megaconial myopathy displayed by dKO mice.

Our previous findings demonstrated a role for Pak1/2 in myoblast differentiation in postnatal development, and this role was reprised during muscle regeneration in response to acute injury [18]. Expression levels of Pak1 and Pak2 were high during perinatal myogenesis, down-regulated in early adulthood, and transiently induced following injury [18]. We hypothesize that Pak1/2 switch from a developmental/pro-differentiation function to a homeostatic function, with the pro-differentiation function called back into play during regeneration. These different functions are likely associated with distinct signaling roles, the former requiring high Pak1/2 levels and exerted mainly through the activation of p38α MAP kinase and the latter achieved at lower Pak1/2 levels and yet to be identified pathways.

The development of skeletal muscle disease in dKO mice raises the possibility that mutations in *PAK1* and/or *PAK2* may underlie human dystrophies or congenital myopathies. We consider this to be unlikely, based on genetic analysis of *Pak1* and *Pak2* in mice. Loss of both *Pak1* and *Pak2* in the muscle lineage is required to produce myopathy, and germline mutations in *Pak2* result in early embryonic lethality [58]. This combination of redundancy and non-viability makes a simple autosomal recessive mechanism analogous to *CHKB* hard to envisage. However, variants that alter expression levels of *PAK1* and/or *PAK2* in humans could contribute to muscle disorders as modifier loci. Expression quantitative trait loci (eQTLs) identify genetic regulation of gene expression and can therefore help link genetics and disease. To examine the potential importance of our *Pak1/2* findings in mice to human disease, we mined the Genotype-Tissue Expression (GTEx) portal (https://www.gtexportal.org/home/) for significant eQTLs within available human tissues for these genes. Skeletal muscle had the greatest number of eQTLs for *PAK2*, but not *PAK1*, with the majority of *PAK2* eQTLs showing downregulation of its expression (Additional file 3: Figure S3 and Additional file 4: Figure S4).

Our use of conditional mutagenesis revealed group I Pak activity as essential for skeletal muscle homeostasis and, unexpectedly, that its loss resulted in a myopathy with distinctive features resembling mice and patients with loss of choline kinase β. Despite their ubiquity as regulators of biological processes, few protein kinases are known to be required for adult muscle homeostasis. Many protein kinases play major roles in development, and their mutation in mice results in embryonic lethality. Conditional mutagenesis is required in such cases; a situation similar to *Pak1/2* is seen with mutation of *Mtor*, which is early embryonic lethal when removed from the germline but results in myopathy when removed selectively from skeletal muscle [59–61]. As central regulators of myriad biological processes, identification of such kinases opens new avenues for understanding skeletal muscle maintenance and possible interventions in muscle disease.

## Conclusions

Loss of group I Pak protein kinases from the skeletal muscle lineage resulted in a late-onset megaconial myopathy. This unexpected phenotype resembles the myopathies associated with loss of Chkβ in mice and patients. Despite this similarity, Pak1/2 and Chkβ activities were not interdependent in mouse skeletal muscle, suggesting a more complex relationship in regulation of mitochondria and muscle homeostasis. Protein kinases are major regulators of most biological processes but few have been implicated in muscle maintenance or disease. Protein kinases are tractable factors for analysis, so further analysis of these group I Paks should provide new insights into these processes.

## Additional files

Additional file 1: **Figure S1.** H&E-stained sections of TA and quadriceps muscles from 1-year-old control and dKO animals. (TIF 667 kb)

Additional file 2: **Figure S2.** Young adult dKO mice show limited signs of muscle disease. H&E-stained sections of uninjured TA muscle from 2-month (2MO) dKO animals showing small, isolated areas of diseased fibers: central nuclei (open arrow), necrosis (closed arrowhead), hypertrophy (asterisk), and fiber splitting (closed arrow). (TIF 1382 kb)

Additional file 3: **Figure S3.** Effect size and distribution of eQTLs for *PAK1*. Jitter plots with overlaid violin plots showing distribution of eQTLs for *PAK1* and their normalized effect size across all tissues sampled. Normalized effect size is computed as the effect of alternative alleles relative to the reference allele in the human genome reference GRCh37/hg19, which is set to 0. (TIF 604 kb)

Additional file 4: **Figure S4.** Effect size and distribution of eQTLs for *PAK2.* Jitter plots with overlaid violin plots showing distribution of eQTLs for *PAK2* and their normalized effect size across all tissues sampled, as in Additional file 3: Figure S3. Note that the skeletal muscle was the tissue with the majority of eQTLs for *PAK2* but not *PAK1*. (TIF 335 kb)

## Abbreviations

CHKB/Chkb: Choline kinaseβ
COX: Cytochrome oxidase
EBD: Evan’s blue dye
EDL: Extensor digitorum longus
MDCMC: Megaconial congenital muscular dystrophy
NADH-TR: NADH-tetrahydrozolium reductase
Pak: p21-activated kinase
PC: Phosphatidylcholine
pCholine: Phosphocholine
PMFDM: Proximal myopathy with focal depletion of mitochondria
TA: Tibialis anterior
