# Supplementary figures and images for: Late-onset megaconial myopathy in mice lacking group I Paks
Source: Skelet Muscle. 2019 Feb 21;9:5. doi: 10.1186/s13395-019-0191-4 (PMC6383276; doi:10.1186/s13395-019-0191-4)

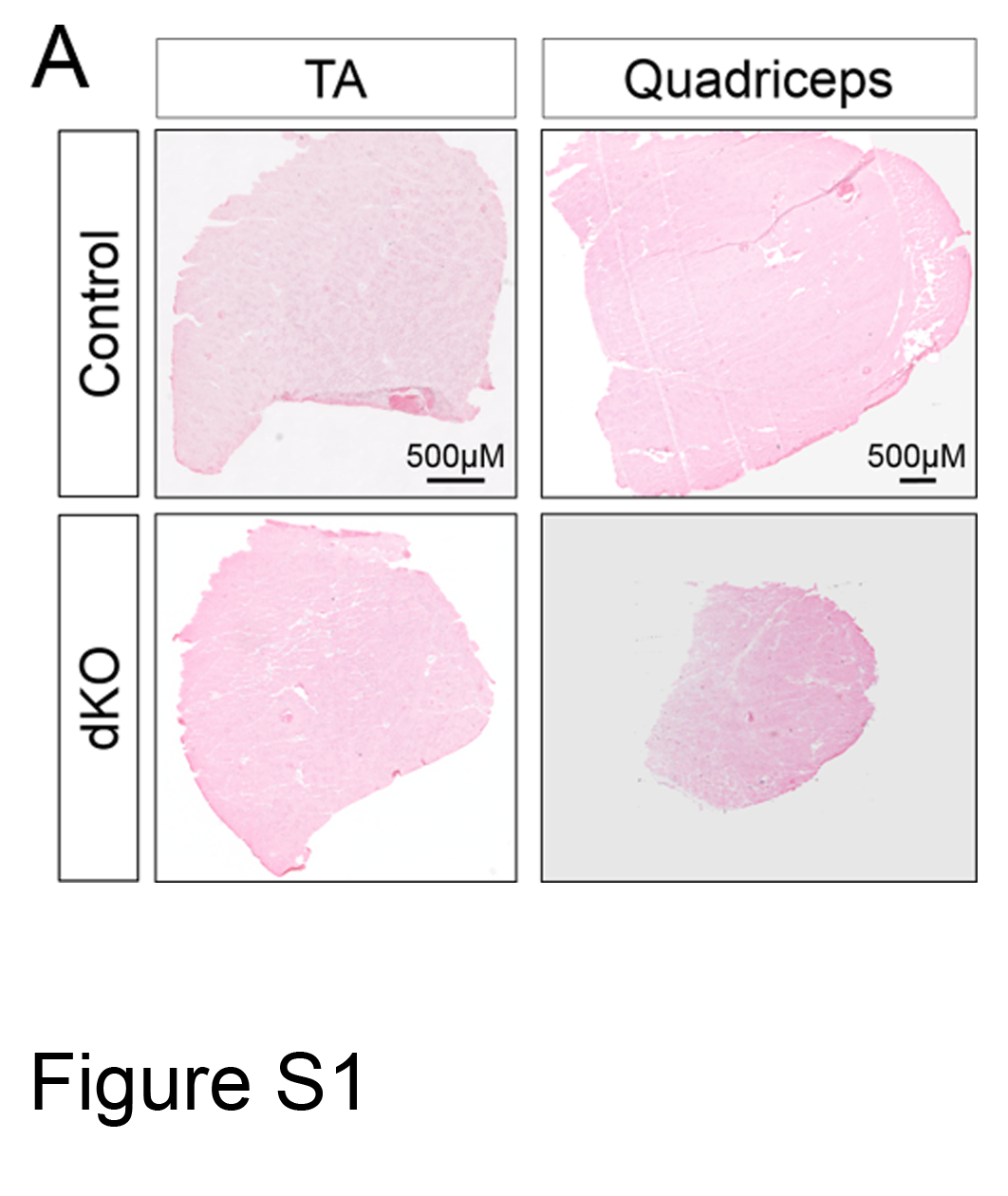

Supplement: Supplementary file 1 — Figure S1. H&E-stained sections of TA and quadriceps muscles from 1-year-old control and dKO animals. (TIF 667 kb) [file 13395_2019_191_MOESM1_ESM.tif]

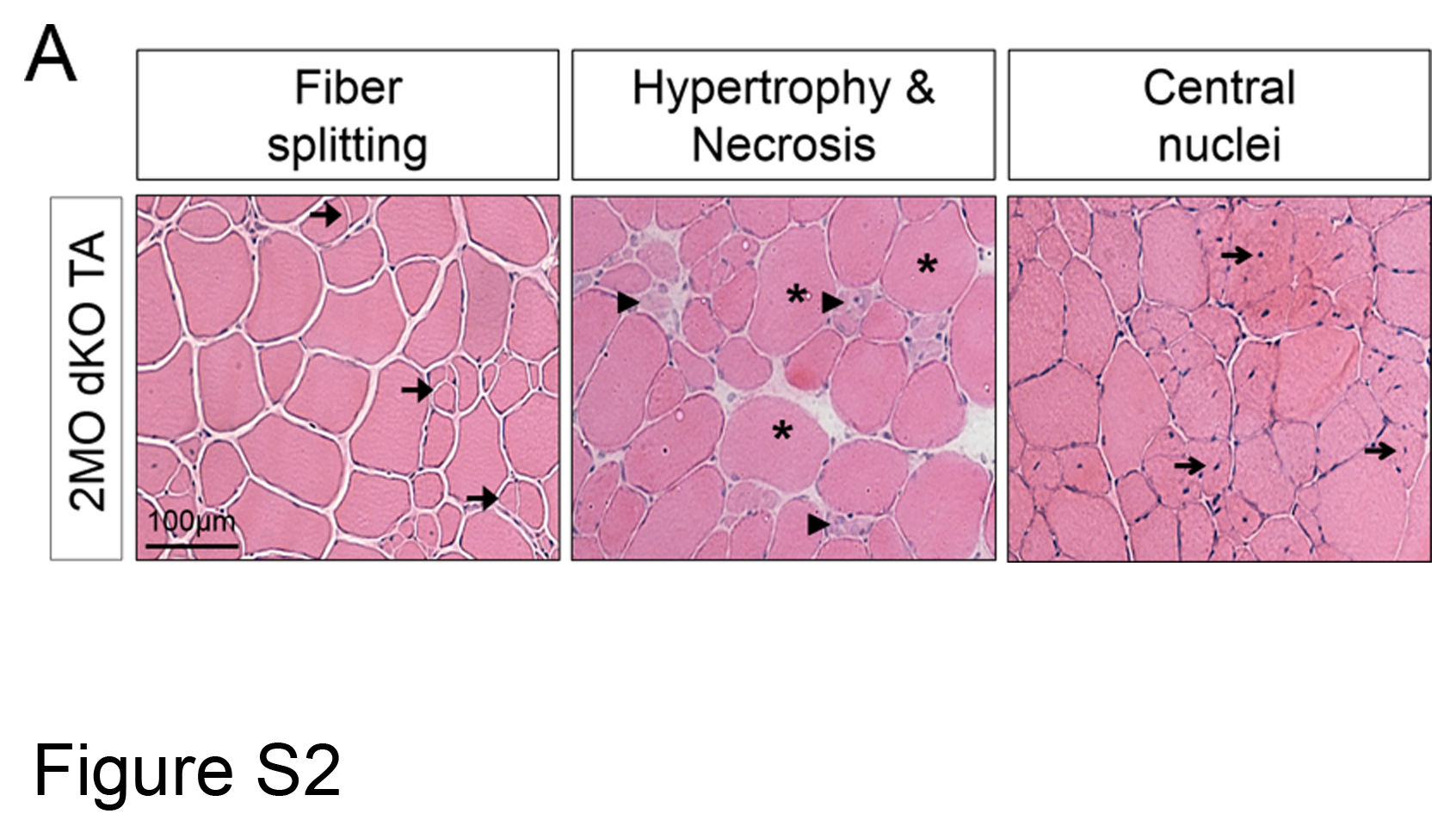

Supplement: Supplementary file 2 — Figure S2. Young adult dKO mice show limited signs of muscle disease. H&E-stained sections of uninjured TA muscle from 2-month (2MO) dKO animals showing small, isolated areas of diseased fibers: central nuclei (open arrow), necrosis (closed arrowhead), hypertrophy (asterisk), and fiber splitting (closed arrow). (TIF 1382 kb) [file 13395_2019_191_MOESM2_ESM.tif]

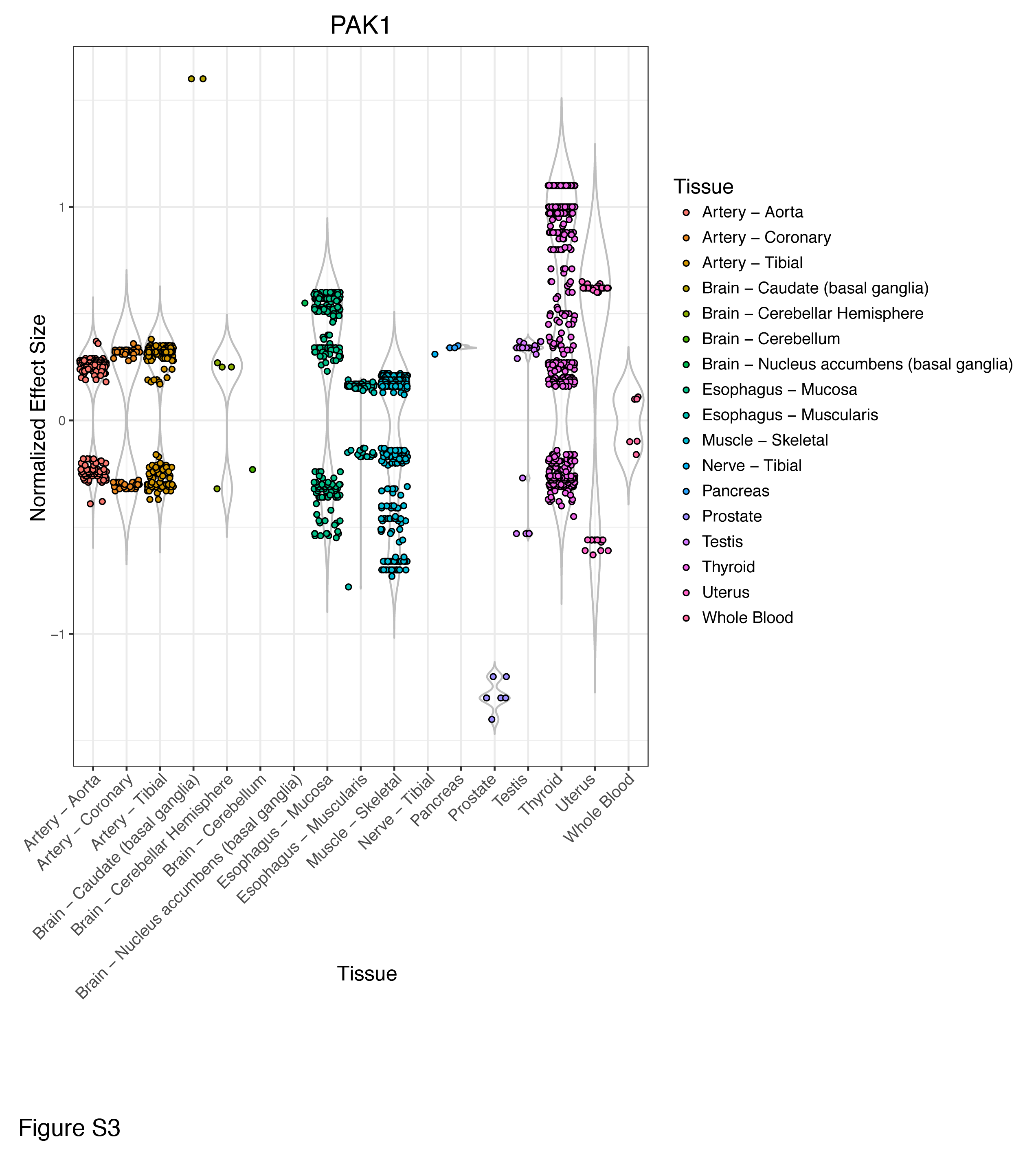

Supplement: Supplementary file 3 — Figure S3. Effect size and distribution of eQTLs for PAK1. Jitter plots with overlaid violin plots showing distribution of eQTLs for PAK1 and their normalized effect size across all tissues sampled. Normalized effect size is computed as the effect of alternative alleles relative to the reference allele in the human genome reference GRCh37/hg19, which is set to 0. (TIF 604 kb) [file 13395_2019_191_MOESM3_ESM.tif]

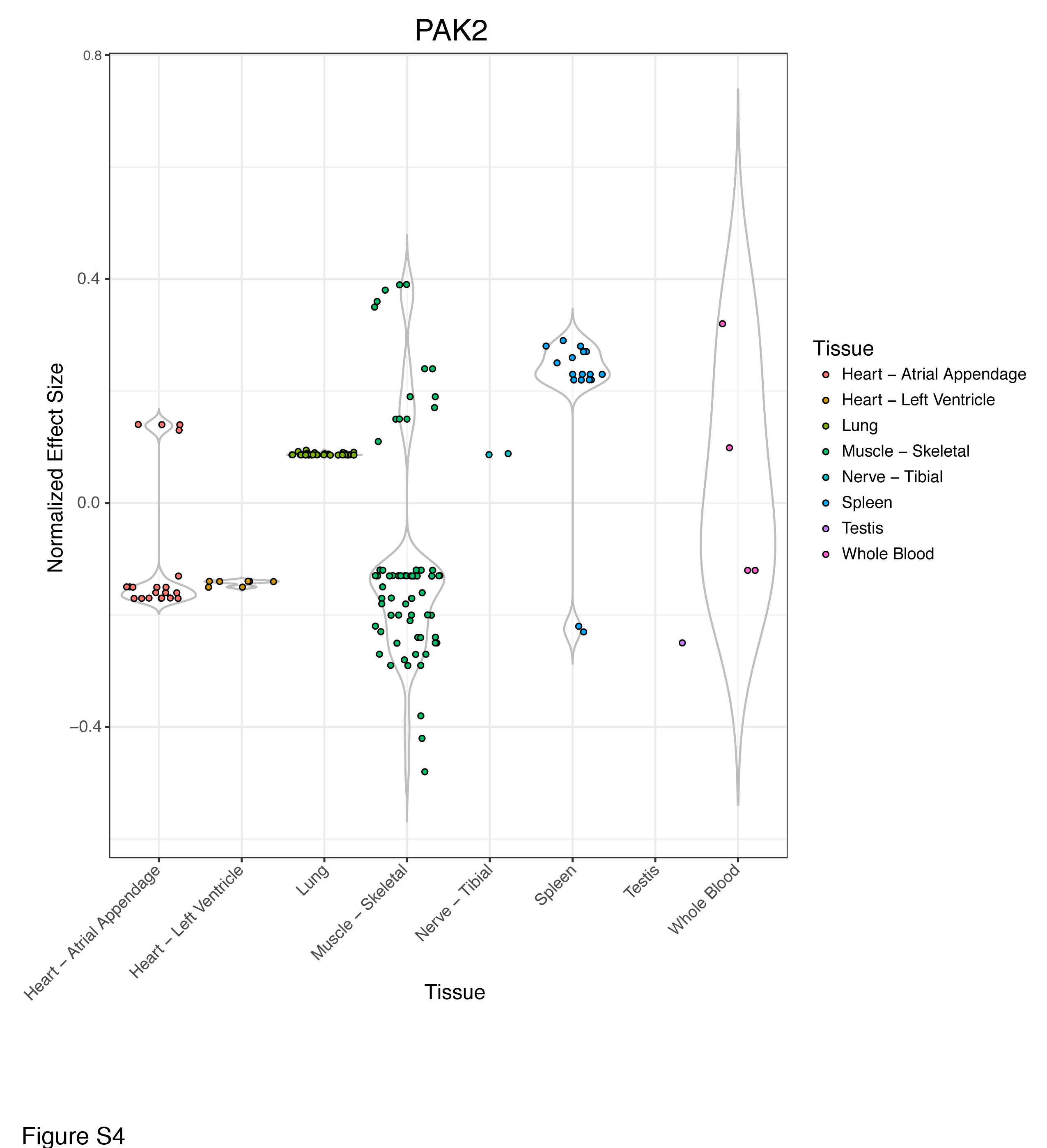

Supplement: Supplementary file 4 — Figure S4. Effect size and distribution of eQTLs for PAK2. Jitter plots with overlaid violin plots showing distribution of eQTLs for PAK2 and their normalized effect size across all tissues sampled, as in Additional file 3: Figure S3. Note that the skeletal muscle was the tissue with the majority of eQTLs for PAK2 but not PAK1. (TIF 335 kb) [file 13395_2019_191_MOESM4_ESM.tif]
